# Den site selection by male brown bears at the population’s expansion front

**DOI:** 10.1371/journal.pone.0202653

**Published:** 2018-08-30

**Authors:** Ane Eriksen, Petter Wabakken, Erling Maartmann, Barbara Zimmermann

**Affiliations:** Department of Forestry and Wildlife Management, Inland Norway University of Applied Sciences, Evenstad, Norway; Université de Sherbrooke, CANADA

## Abstract

Brown bears (*Ursus arctos*) spend about half of the year in winter dens. In order to preserve energy, bears may select denning locations that minimize temperature loss and human disturbance. In expanding animal populations, demographic structure and individual behavior at the expansion front can differ from core areas. We conducted a non-invasive study of male brown bear den sites at the male-biased, low-density western expansion front of the Scandinavian brown bear population, comparing den locations to the available habitat. Compared to the higher-density population core in which intraspecific avoidance may affect den site selection of subordinate bears, we expected resource competition in the periphery to be low, and all bears to be able to select optimal den sites. In addition, bears in the periphery had access to free-ranging domestic sheep during summer. We found that males in the periphery denned on high-elevation slopes, probably providing good drainage, longer periods of consistent, insulating snow cover and fewer melting-freezing events. Forests were the principal denning habitat and no dens were found in alpine areas. The Scandinavian brown bears have a history of intense harvest, including culling at the den. This may have exerted a selection pressure to avoid denning in open alpine habitat which compared to forests provide little cover. The bears denned away from main roads and in steep, rugged terrain, probably limiting human access. The odds for finding a bear den decreased with increasing distance to the population core where females could be found. Previous studies have documented directed movement of male brown bears from the male-biased population periphery toward the core areas during the mating season. In this way, denning males may be trading off between low resource competition and access to sheep in the low-density periphery, and mating opportunities in the higher-density population core.

## Introduction

In areas of high latitude, the winter season presents animals with the challenges of low temperatures and reduced food availability. Animals show a range of physiological and behavioral adaptations to these conditions, such as migration [1], food caching [2], metabolic rate reduction [3], seasonal changes in fur, plumage, and fat deposits [3, 4], and hibernation [5]. Across much of their Holarctic range, brown bears (*Ursus arctos*) spend 5–7 months of the year in winter dens [6–9]. During hibernation, they lose 20–45% of their body weight [10], and selecting a den site that minimizes energy loss is therefore essential [7]. Furthermore, dens provide cover for security [11]. Through history, denning bears have been vulnerable to humans [12–15]. In Scandinavia during the 1800s, culling at the den was one of the most common bear hunting methods as it was considered the most effective way of reducing bear numbers [13, 14]. In addition, den abandonment due to human disturbance has substantial energetic costs and has been linked to increased cub mortality [15–20]. Therefore, bears may select den sites that minimize human disturbance [21–24].

**Fig 1:**
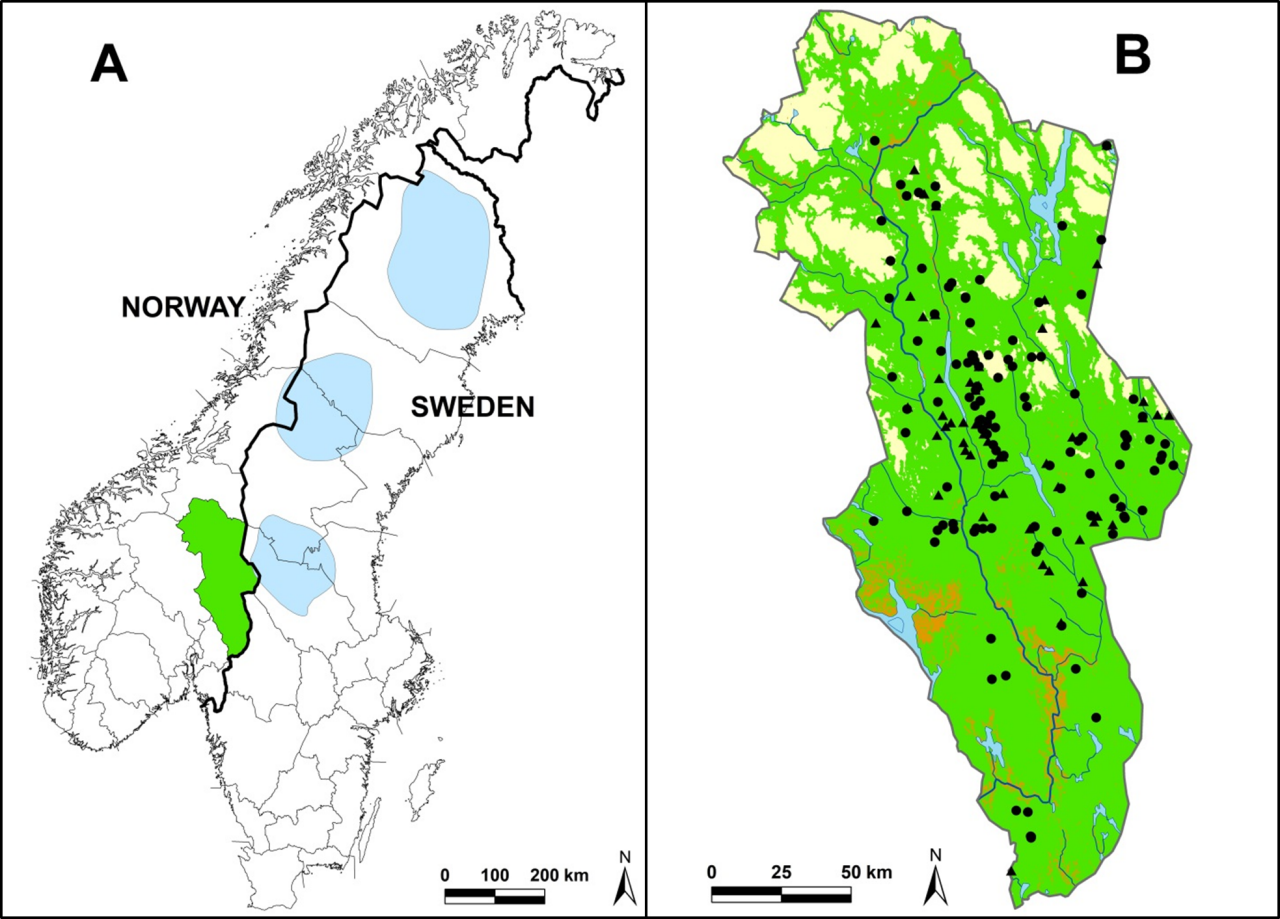
Study area. (A) Location of the study area Hedmark County in South-Eastern Norway (green) and brown bear population core areas (blue) [38] on the Scandinavian Peninsula. (B) Hedmark County with major cover types forest (green), alpine (yellow), cultivated land (brown), lakes (blue), rivers (black lines), and the location of 174 dens used by brown bear males from 1976 to 2014. Black dots represent 120 dens used by 64 different males identified by DNA, and black triangles represent 54 dens used by unidentified males (see Methods).

In expanding animal populations, population structure, genetic diversity and individual behavior at the expansion front can differ from core areas [25–34], and hence, findings from population cores may not be transferrable to peripheral areas. Such demographic and behavioral differences have been found in expanding brown bear populations in different parts of Europe [31–36]. The Scandinavian brown bear population has expanded considerably after being functionally extinct in Norway by the 1940s and reduced to a population low of about 130 individuals in several small, isolated areas in Sweden, by 1920–30 [30, 37]. The dispersal is male-biased, whereas the females reside mostly within defined population core areas, still almost exclusively on the Swedish side of the national border (Fig 1A) [38–41]. South-Eastern Norway is now at the population’s expansion front, and like peripheries of other expanding brown bear populations [31–33], consisting primarily of males dispersing from the core areas [30, 37, 41] to which they periodically move back for the mating season, presumably to get access to females [42]. Even though some bears in South-Eastern Norway are shot in association with livestock depredation and in later years during licensed hunts, the number of bears shot per year is low. For more than 100 years and until after our study period, no female bears were confirmed killed in this peripheral area [43]. The habitat quality is similar along the core-periphery gradient [44], except for free-ranging domestic sheep which are common in Norway during summer and early fall, but not available in Sweden [42, 45, 46]. Hence, the low density and male-skewed sex ratio west of the national border are results of the male-biased population expansion, and not due to harvest or unsuitable habitat [30, 37, 44]. On the contrary, sheep provide an additional source of lipids and proteins for bears in the periphery.

Denning behavior and selection of denning habitat have been investigated previously for Scandinavian brown bears in the southernmost population core area (Fig 1A) [9, 24, 47, 48]. In this area, bears most often dig their dens into anthills or soil, or hibernate on a nest of sticks and branches on the ground, or under rocks [9, 48]. Males have been reported to enter their dens between 5 October and 18 November (mean = 27 October), possibly triggered by the first snowfall, and leave their den before the snow has melted, between 6 March and 25 April (mean = 4 April) [9]. Bears in the core area have been found to prefer den sites in open canopy pine forest, at lower altitude, easterly aspect and steep slopes, and to avoid roads, water, peat, exposed bedrock, and deciduous and alpine mountain-birch forests [47]. However, there has been no den site selection study in the periphery of this population, and hence it is not known whether individuals at the expansion front show similar denning preferences to those in the higher bear-density core areas. In the population core, intraspecific avoidance may affect den site selection of females and subadult males which were found to den closer to occupied houses and plowed roads, possibly to avoid adult males [47, 48]. Also, the preference for lower altitudes in the population core may be attributed to avoidance of dominant males as it was evident only in females and subadult males [47, 48]. In the periphery, home range sizes increase as the bear density drops, but without a decrease in habitat quality [44]. Hence, intraspecific interactions and resource competition should be low [36, 44], and even young males may be able to select optimal den sites. The low competition and access to sheep in the periphery may compensate for the travel distance to the core areas during the mating season, especially for young males.

By non-invasive field methods, we conducted the first study of winter dens used by brown bear males at the expansion front of the Scandinavian brown bear population, within Hedmark County in South-Eastern Norway. We expected that brown bear males in this periphery could optimize their den site selection to maximize heat preservation and minimize human disturbance without having to trade-off with intraspecific avoidance. To preserve heat, we hypothesized that bears in the periphery would den at high elevation (H1) and avoid southerly aspects and high potential solar radiation (H2), thus providing a longer period of consistent, insulating snow cover and fewer melting-freezing events [49, 50]. We also hypothesized that denning bears would avoid water and peatland, and den on slopes for good drainage (H3). To minimize human disturbance, we hypothesized that dens would be located preferentially in steep, rugged terrain to limit human access (H4), in forested areas providing shelter (H5), and away from human structures (H6). Finally, we hypothesize that males in the periphery would prefer to den relatively close to the population core to facilitate access to females (H7).

## Study area

Hedmark County in South-Eastern Norway (27 397 km^2^, 61°N, 11°E) is at the expansion front of the Scandinavian brown bear population, west of the southernmost population core area (Fig 1A). The county borders Sweden to the east and has by far the highest concentration of bear dens in Southern Norway, i.e. 185 out of 205 used bear dens located non-invasively in Norway during the 42-year study period 1973–2014 (Fig 1B). During this period, the bear density in Hedmark was low (< 1 bear/1000 km^2^) [51], predominantly consisting of males dispersing from the population core areas [30, 37]. Out of the 22 municipalities that make up Hedmark County, throughout the study period female brown bears were only confirmed in the eastern halves of the two municipalities closest to the bordering Swedish core area, except for one dispersed female and her one female offspring during the last three years of our study. During the study period, 51 brown bears were shot in Hedmark [43]. All of these were males, and were killed in association with attacks on livestock (N = 32), during licensed bear hunts (N = 13, the first one in 2007), shot illegally (N = 4), or in self-defense (N = 2). In addition to brown bears, the large carnivore community in Hedmark includes breeding wolves (*Canis lupus*), lynx (*Lynx lynx*) and wolverines (*Gulo gulo*) [45].

Hedmark is dominated by boreal forests (59%), alpine mountain ranges (16% above the tree line), peatland (11%), and agricultural areas (4%). The topography of the northern part is made up of parallel hills and river valleys running generally from north to south, with alpine areas reaching as high as 2 178 m a.s.l. (Fig 1B). The southern part of the county is of generally lower elevation. The climate is continental with temperatures ranging from 30°C in summer to -40°C in winter, although average temperature in January is more moderate, from -6 to -8°C in the southern areas, and from -10 to -13°C in the northernmost valley areas. The tree line ranges from 750 to 1 050 m a.s.l. Up to approximately 750 m a.s.l., the vegetation is dominated by boreal coniferous forest, with Scots pine (*Pinus sylvestris*) and Norway spruce (*Picea abies*) as the dominating species. Above the conifer tree line is the subalpine birch zone above which alpine vegetation takes over. Within the forested areas, the winter snow cover is deeper at higher elevations.

Commercial logging has resulted in an extensive network of forest gravel roads (0.68 km/km^2^). Average public road density is 0.24 km/km^2^, and the major roads are found in the valley bottoms. The human population density is low, with an average of 7.13 persons/km^2^ in late 2016, but about half the population lives in settlements covering 0.2% of the area. The average rural density is 3.1 persons/km^2^. Recreational cabins of different standard and size are widely distributed throughout the study area with an overall density of 1.34/km^2^. In fact, seven out of 22 municipalities have a larger number of recreational cabins than inhabited houses. The majority of cabins are clustered in association with alpine winter sport areas near the tree line.

Hedmark is the county in Norway with the most extensive moose hunt. Each year, during the hunting season which overlaps with the period of brown bear den entry, 6000–7000 moose hunters and a comparable number of small game hunters are active in the area. As well as being a major game species, moose are an important food resource for brown bears in spring [46, 52–54]. Moose are available throughout the core and periphery of the Scandinavian brown bear population [44], with densities estimated at 1.3–3.4 moose/km^2^ in central Hedmark in 2003 and 2004 [55]. During summer and early fall (generally between 1 June and 10 September) throughout the study period, brown bears in Hedmark had access to free-ranging domestic sheep with an average density of 4.9 sheep/km^2^ in 1990–1992 [42]. Free-ranging sheep are not found in the Swedish core area [46]. As the sheep are collected 1–2 months before brown bear den entry, we do not expect the distribution of sheep within the study area to affect den site selection.

## Methods

### Non-invasive collection of denning data

Brown bear den monitoring was initiated in Southern Norway, including Hedmark County, in 1973, when the bear was legally protected in Norway. The first den location was confirmed post-hibernation in spring 1976. In this study, we include locations of dens used by brown bear males in Hedmark from the winters of 1975/1976 through 2013/2014 (S1 Fig). Tracks from recently emerged bears were found randomly by us or reported by others, and we subsequently located the dens by backtracking on snow. All bear tracks were backtracked as long as possible in search for dens. In addition, some dens were found by chance without tracking, either by us or reported by others (hunters, skiers, hikers, forestry workers, media etc.). Although a few bears wore radio collars for other research projects [34, 42], all dens were found without the use of telemetry, and samples collected by us after the bears had left the area. Hence, we did not trap or otherwise disturb bears as part of our study. As the data sampling was non-invasive without the use of live animals, the study did not require an ethical permit. Only bear dens that we could confirm had been used for hibernation were included. Dens completely excavated, but with no bedded lair and hair from the bear, were excluded. A total of 185 dens were confirmed used and inspected in the field, either by us (N = 137) or by trained field personnel under our guidance (N = 48). From 1996 and onwards, non-invasive DNA samples from hair and feces were collected at the den site for sex and ID determination. The DNA samples were analyzed at the NIBIO (Norwegian Institute of Bioeconomy Research) lab by methods described elsewhere [56], and in most cases publicly available in the national large carnivore database [43]. In addition, the identity was known for a few bears radio-collared prior to 1993 [34]. Some additional bears were classified as males by a combination of leaving the den very early in spring, large track size, and den location far outside the female core area. As a result, 174 dens were concluded to have been used by males, and among these 120 were used by 62 different males of known identity (S1 Fig). The age was known for a few radio-collared bears [34, 42]. In addition, for DNA-identified denning bears that later were killed during hunting, the age was estimated by counting the annuli of a premolar tooth according to Matson, van Daele [57]. We obtained detailed information about how the dens were found for 165 of the male dens, including the location of first detection (i.e. where the tracks leading to the den were found, or the den itself when found directly without tracking). In 122 cases (74%), the dens had been found by backtracking on snow in spring, including 92 dens used by 53 different males of known identity.

### Habitat variables

We mapped the habitat in the study area using national topographic maps (1:50’000 N50-maps, Norwegian Mapping Authorities). Spatial analyses were performed using ArcGIS 10.3 (ESRI, Redlands, CA). The habitat variables are summarized in Table 1.

**Table 1:** Habitat variables used to study den site selection of brown bear males in Hedmark County.

Category	Variable	Description	Use
Cover type/ topography	Elevation zones	Categorical; *alpine*: all areas above the tree line area; *tree line area*: the area within 200 m on either side of the border line between forest cover and open mountain areas, *below tree line*: all areas below the tree line area, *non-alpine*: tree line and below tree line areas combined	Cover type analysis
	Cover type	Categorical; *forest*, *peatland*, *cultivated land*, *settlements*, *water*	Cover type analysis
	Residual elevation	Continuous; deviation (m) from the elevation predicted from the latitude based on the model *elevation ~ UTM Northing*.	Habitat model
	Slope	Continuous; degrees. Calculated using a digital elevation model with raster cell size 250 m, transformed by log(x+1)	Habitat model
	Aspect	Categorical; *N*: 315°-44°, *E*: 45°-134°, *S*: 135°-224°, *W*: 225°-314°. Calculated using a digital elevation model with raster cell size 250 m	Habitat model
	Ruggedness	Index ranging from 0 (flat) to 1 (most rugged) [58]. Calculated using a digital elevation model with raster cell size 250 m, transformed by log(x+1)	Habitat model
	Solar radiation	Continuous; maximum possible, cumulative solar radiation (kWh/m^2^) 1 November—1 April. Calculated using a digital elevation model with raster cell size 250 m	Habitat model
Disturbance	Distance to main road	Continuous; shortest distance (km) to public road, transformed by log(x+1)	Habitat model
	Distance to forest road	Continuous; shortest distance (km) to private road, transformed by log(x+1)	Habitat model
	House density	Continuous; kernel density rasters, 250 m cell size, 5 km search radius, transformed by log(x+1)	Habitat model
	Cabin density	Continuous; kernel density rasters, 250 m cell size, 5 km search radius, transformed by log(x+1)	Habitat model
Population core	Distance to population core	Shortest distance (scaled to 10 km) to the main distribution range of reproducing females [59]	Habitat model

#### Cover types and topography

We digitized the tree line as the border between forest cover and open mountain areas based on the N50- maps, and defined the tree line area as the area within a 200 m buffer on either side of this line. The area above the tree line area was considered alpine, and the area below it was considered below tree line. The below tree line and tree line areas combined were considered non-alpine. We used the N50-maps to assign cover type to each den location. We used a digital elevation model with 250 m raster cell size to calculate slope, aspect, a ruggedness index [58], and the maximum possible, cumulative solar radiation in the period 1 November to 1 April (Table 1).

#### Disturbance

We mapped all the roads in the study area from the N50-maps. Roads were categorized as either main roads (public, mostly paved and connecting human settlements), or forest roads (private, largely unpaved, created for logging and forest management). Individual buildings were categorized into houses (N50 building type no. 111–113, 121–124,131–136, 141–142, 151–152, 159) and recreational cabins (no. 161–163, 171). Buildings within settlements are not registered as single points in N50-maps, but as polygons delineating the built-up areas. We therefore created artificial buildings in those polygons by generating regularly distributed points with a density of 1 point/1000 m^2^. The combined real and artificial buildings were turned into density rasters of 250 m cell size by applying the kernel density tool in ArcGIS 10.3 with a search radius of 5 km (Table 1).

#### Distance to population core

The main distribution range of reproducing brown bear females in Scandinavia has expanded from approximately 40 km east of our study area in the 1980s, reaching the national border between Sweden and Norway 10–20 years later [30, 34, 35, 38]. Annual updates of the population core area are not available. In the current study, we use the core area given in 2006 by Sahlén, Swenson [59], which is the closest available to the median (2003) and mean (2002) year for the dens in our study.

### Study design and statistical analyses

#### Major cover types

Based on our hypothesis that forest is the predominant cover type for bear dens in Hedmark, we first ran three analyses to identify broad, major cover types across the entire study area as either non-habitat or habitat for bear denning. In these analyses we compared the observed number of dens in different habitat categories to the expected number based on the proportion of the study area covered by the respective habitat category. We first compared observed and expected use of (1) alpine vs. non-alpine areas. Within the non-alpine areas, we then compared (2) areas within vs. below the tree line area, and (3) cultivated land, peatland, open areas, settled areas, forested areas, and water. These initial analyses allowed us to avoid multiple-level categorical cover type variables in the habitat models.

#### Den site habitat models

We compared den locations to all available habitat within the 27 397 km^2^ study area. Mean annual home range size of Scandinavian brown bear males is approximately 1 500 km^2^ in the southernmost population core area [34]. However, home range sizes increase with decreasing population densities [44]. In low-density peripheral areas, such as our study area in which males move towards the core areas during the mating season [42], annual home ranges can be as large as 28 000 km^2^ [34, 42]. Natal dispersal distance of most brown bear males in Scandinavia is between 100 and 140 km (mean = 120 km), and distances up to 467 km have been observed [60]. Hence, given the low bear density at the population periphery and the potential for overlapping home ranges [61, 62], our entire study area should theoretically be available to males dispersing west from the southernmost population core area (generally at 2–3 years of age; [60]).

We used logistic generalized linear mixed models (GLMMs) and created resource selection functions (RSFs) [63] to compare the 120 den sites used by 62 males of known identity (used locations) to 1000 random points generated within the study area (available locations). No random points were created in water, settlements, on cultivated land or in alpine areas as these were regarded as non-habitat for bear denning (see Results). To control for pseudoreplication, we included bear ID as a random predictor. Each random point was assigned a bear ID from a randomly selected den, giving 8–9 random points per den.

Because there was a general increase in elevation from south to north in the study area (*R*^*2*^ = 0.64, *p* < 0.0001), we did not use elevation directly in the models, as the latitude, and hence the elevation of the bear dens could be affected by the latitude of the bears’ place of origin in the population core. Instead, we calculated the residuals from the predicted elevation based on a linear model using the 1000 random points, with elevation as the response variable and the UTM northing as the single predictor. This deviation from the predicted elevation was labeled “residual elevation” and used in the models. Thus, the explanatory variables used in the full model were residual elevation, slope, aspect, ruggedness, solar radiation, distance to nearest main road and forest road, density of houses and cabins, and distance to the border of the closest population core area (Table 1). Residual elevation, slope, ruggedness, solar radiation and distance to roads were included with quadratic terms in the full model, as bears might select for intermediate values for these predictors.

Before fitting the full model, we calculated correlation coefficients for all the predictor variables and avoided using pairs of variables with |*r*| > 0.7 [64]. We also calculated the variance inflation factor (*VIF*) of the predictor variables using the car package in R [65], and avoided using variables with *VIF* ≥ 5. The most parsimonious models were then selected using a drop 1 stepwise procedure based on the Akaike Information Criterion (*AIC*). Among the best fit models (*ΔAIC* < 2) we selected the simplest one.

To validate the selected model, we performed k-fold cross-validation according to Boyce, Vernier [66]. The data were partitioned into a validating set containing the dens of 10 randomly selected bears and the corresponding random points, and a training set containing the remaining 52 bears and the corresponding random points. We ran the model using the training set, extracted the model coefficients of the fixed effects and used them to predict the RSF values of the data points in the validation set. The validating set was then sorted by the RSF value and split into 10 equal-sized bins. For each bin we calculated the relative frequency of used points. We calculated the Spearman rank correlation coefficient (*r*_*s*_) between the rank of the bin and the relative frequency of used points as an indicator of model fit. We repeated this process 100 times and calculated the average *r*_*s*_ as an indicator of the model’s predictive power.

#### Probability of detection

Because the dens or tracks leading to the dens were found opportunistically, the probability of detection may have been biased towards areas with easy access near roads or human settlements. To evaluate the likelihood of such a bias, we used logistic GLMs comparing the 165 locations of first detection (where the tracks or dens were initially found) to the available habitat represented by the 1000 random points. The explanatory variables included in the full model were residual elevation with a quadratic term, slope, ruggedness, distance to main road and forest road, and density of houses and cabins. The model selection process was identical to the den site habitat models.

We used an alpha level of .05 for statistical tests. Statistical analyses were performed in RStudio version 0.99.902 [67] running R version 3.3.0 [68].

## Results

During the 42-year study period, 185 dens were located independently of telemetry in Hedmark County. Among these, 174 dens (94%) were used by males and thus included in this study, and 120 were used by 62 different males of known identity (S1 Fig). The excluded dens were either confirmed used by females by DNA or tracks of females with young (N = 9), or used by bears of unknown sex (N = 2). Mean number of dens per identified male was 1.89 (range = 1–12). Only two of the dens were used twice, and these were both re-used by the same bears that used them the first time. Most of the male dens (92%) were either anthill dens or open nest dens, whereas the remaining 8% were rock or soil dens. Throughout our study, none of the denning bears of known origin were born in Norway, but had dispersed across the border from Sweden. The age was known for 26 of the 62 identified males. These bears were on average 4.7 years old when emerging from their first registered den in Hedmark (range = 2–10, *SD* = 1.9). When including all dens from the 26 males of known age (N = 55 dens), the average age of the denning bears was 8.2 years (range = 2–25, *SD* = 5.4). Among the 55 dens used by males of known age, 15 were used by subadults (< 4 years of age) and 40 were used by adult males. This sample did not allow us to separate between den site selection of adult and subadult males in the analyses.

### Major cover types

**Fig 2:**
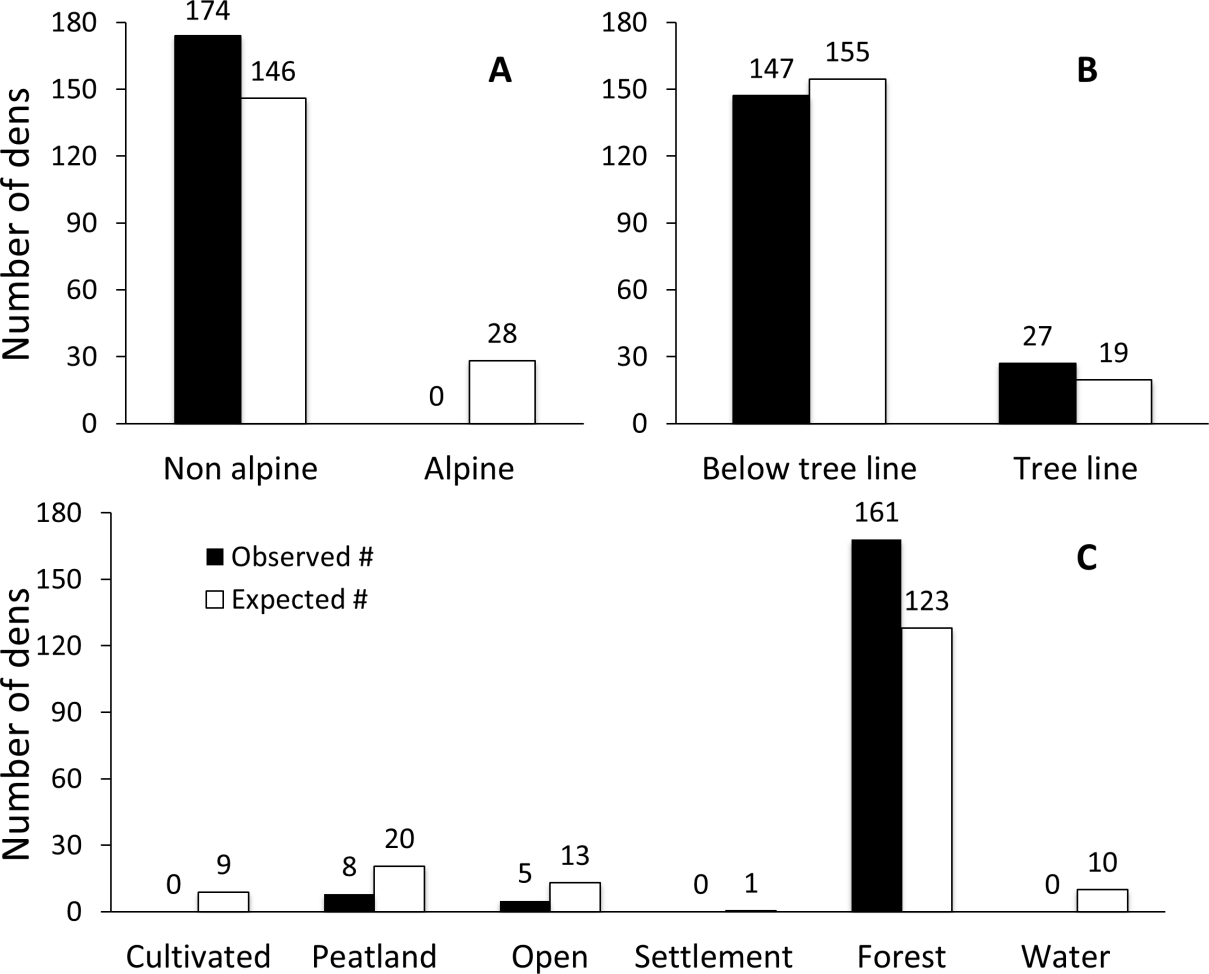
Observed vs. expected number of male brown bear dens in different cover types. Expected numbers were calculated from the proportion of the study area with the respective cover type. Alpine areas were excluded in Fig 2B and 2C.

Among the 174 male dens, 84.5% were below the tree line and 15.5% were in tree line areas. No dens were found in alpine areas. This distribution differed from the availability of habitat. Non-alpine areas were used more than expected from their availability, whereas alpine areas were not used at all, despite constituting 16.2% of the study area (*Χ*^*2*^ = 33.64, *df* = 1, *p* < 0.0001, Fig 2A). We therefore considered alpine areas non-habitat for male brown bear denning in the den site habitat models. Within non-alpine areas, tree line areas were used slightly more and areas below the tree line slightly less than expected, but this was not statistically significant (*Χ*^*2*^ = 3.261, *df* = 1, *p* = 0.071, Fig 2B).

Dens were not distributed randomly across cover types within non-alpine areas (*Χ*^*2*^ = 44.58, *df* = 5, *p* < 0.0001). Forest, covering 70.7% of the non-alpine areas, contained 92.5% of the dens, which is more than expected from its availability (Fig 2C). Peatland and open areas contained 4.6% and 2.9% of the dens respectively, and were both used less than expected from the availability, whereas cultivated land, settlements and water were not used at all, and were therefore considered non-habitat for bear denning in the habitat models (Fig 2C).

### Den site habitat models

**Fig 3:**
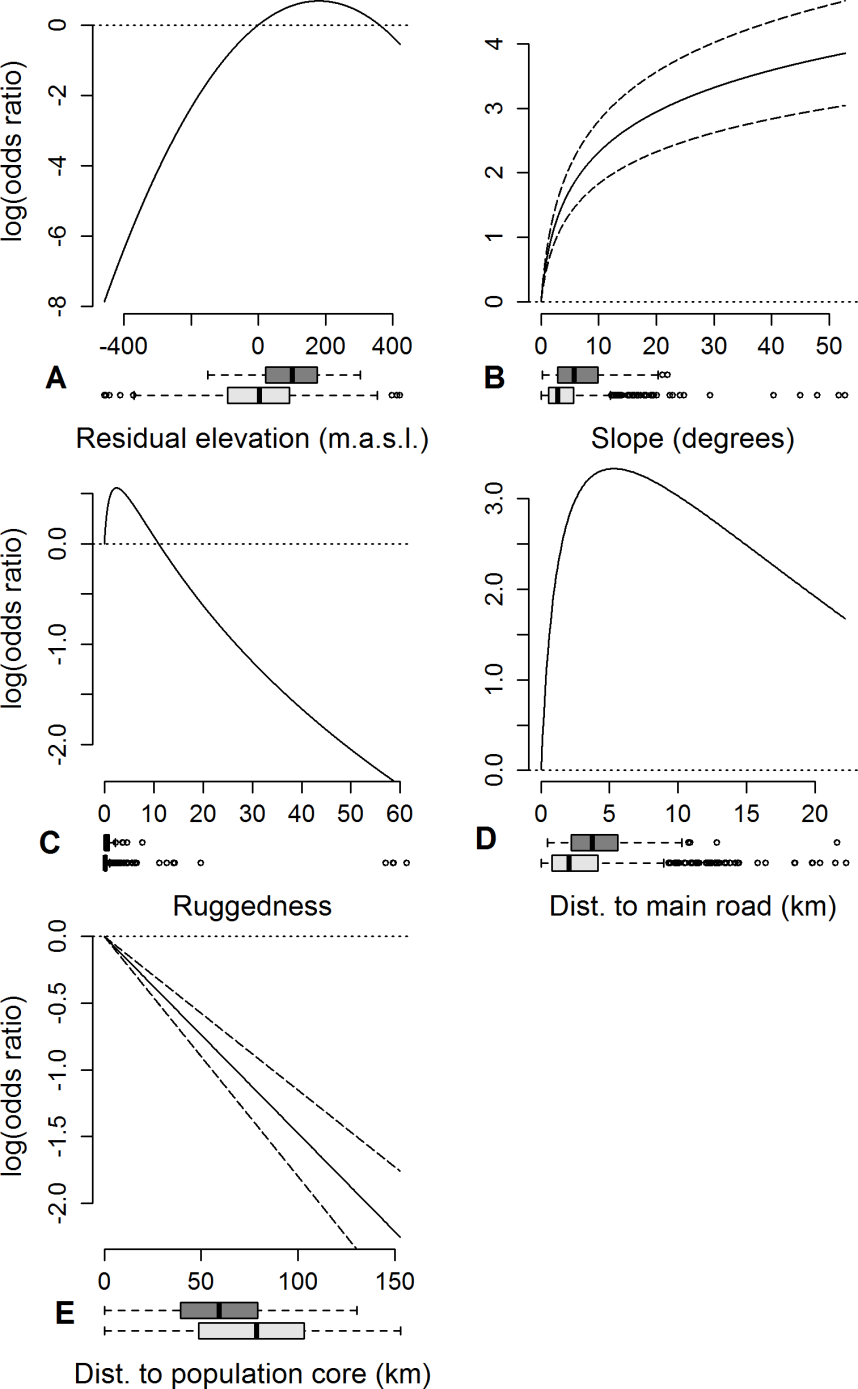
RSF scores of the covariates in the selected logistic GLMM comparing 120 den locations from 62 identified male bears with 1000 random points in Hedmark County. Standard errors are included for linear covariates, and the horizontal dotted lines mark the reference value of log(odds ratio) = 0 intersecting with x = 0. Distributions for den locations (dark grey) and random locations (light grey) are given in boxplots.

All pairs of covariates had |*r*| < 0.7. Furthermore, all covariates had a *VIF* < 2, and were included in the full model. The model selection resulted in four models with indistinguishable *AIC* values (*ΔAIC* < 2, S1 Table). We selected the model with the smallest number of predictors (M9, S1 Table), which had a conditional *R*^*2*^ value (variation explained by both the fixed and random factors) of 0.59. According to this model, the odds for a data point being a den rather than a random point increased with increasing residual elevation, up to 180 m above the elevation predicted from latitude (Table 2; Fig 3A). Mean elevation of den sites was 684 m a.s.l., and the dens were on average 104 m higher than the predicted elevation (Wilcoxon signed rank *V* = 6688, *p* < 0.0001). The odds for a point being a den increased with increasing slope, although no dens were on slopes steeper than 22 degrees, and the model included a quadratic effect of ruggedness (Fig 3B and 3C). Note however that the 95% confidence intervals for the ruggedness estimates did encompass zero (Table 2). The only disturbance parameter included in the selected model was distance to main road. According to the model, the odds for a data point being a den peaked at 5.3 km from main roads (above the third quartile of the distribution of distance to main road in the available habitat, Fig 3D). Finally, increasing distance to the population core areas was associated with lower odds for a data point being a bear den (Fig 3E). The selected model did not include aspect, solar radiation, distance to forest road, or density of houses and cabins (Table 2). The K-fold cross-validation of the selected model gave *r*_*s*_ = 0.86, indicating good predictive power.

**Table 2:** Summary of selected den site habitat model. Logistic GLMM (M9, S1 Table) comparing 120 den locations from 62 individual male brown bears with 1000 randomly generated points within Hedmark County. Bear ID was included as a random effect.

Covariates	Estimate	95% CI
Residual elevation	7.56	3.92–11.21
Residual elevation^2	-21.00	-36.02–-5.98
Slope (log(x+1))	0.97	0.57–1.37
Ruggedness (log(x+1))	0.90	-0.19–1.98
Ruggedness^2	-0.36	-0.86–0.14
Dist. to main road (log(x+1))	3.61	1.74–5.49
Dist. to main road^2	-0.98	-1.57–-0.39
Dist. to population core area	-1.47	-2.11–-0.84

### Probability of detection

The straight-line distance between the den and the location of first detection (where the tracks or dens were initially found) ranged between 0 m (den found directly without tracking) and 20.6 km. According to the selected logistic GLMs comparing the locations of first detection to the available habitat, the locations of first detection were generally at either higher or lower elevation than predicted from the latitude (both linear and quadratic terms positive), steeper slopes, farther from forest roads, and at lower densities of houses and cabins compared to the available habitat. For model selection and model summary, see S2 and S3 Tables.

## Discussion

Our findings support the notion of den site selection in brown bears as a multifactorial process, and suggest that male bears distinguished between suitable and unsuitable habitat. Heat preservation is of great importance to hibernating bears, but higher or variable winter temperatures may result in earlier snowmelt and surface water inside the den [7], and therefore contribute to heat loss. Bears may rather prefer dry sites where temperatures stay consistently below freezing for a longer period [7, 47]. The selected den site habitat model supported our hypothesis of a preference for high elevation (H1), which in our study area is associated with longer periods of consistent snow cover [49]. We found that habitat 180 m above the elevation predicted from the latitude had the highest odds for having a bear den (Fig 3A). That is, at any given latitude, bear dens were generally found up in the hills, and not in the valley bottoms. In contrast, bears in the core area bordering with our study area were found to den at lower elevation [47]. However, adult males in the population core were the exception and showed no such selection [47]. In the population core, females and subadult males may be forced into suboptimal denning habitat at lower elevation to avoid dominant males, whereas in the low-density periphery, even young males may be able to select optimally. Unfortunately, our dataset of dens used by males of known age was too small to compare den site selection of adult and subadult males in the models.

To maximize the insulating snow cover, we hypothesized that bears may prefer the aspect that accumulates the most snow [6, 7] and receives the least amount of sunlight (H2). We found no support for this hypothesis as the selected model included no effects of aspect or solar radiation. More of the dens faced east (N = 56) or west (N = 57) than north (N = 31) or south (N = 30), but this was merely a reflection of the available habitat, as the valleys in the study area generally run from north to south. Preferences for all aspect categories have been found in previous den site selection studies [47].

Avoidance of water and peatland, and a preference for steep slopes for effective drainage would minimize surface water inside the den (H3), and was found in the current study as well as in the southernmost population core area [47]. The bears denned in steeper and slightly more rugged terrain than expected from the available habitat, probably also limiting human access (H4). However, we found no dens in the small fraction of the habitat with the very highest slope and ruggedness values (Fig 3B and 3C).

During our study period, the Hedmark bears predominantly denned in forest; the only cover type used more than expected from its availability, hence supporting our hypothesis H5. No dens were found in alpine terrain, above the tree line buffer area. We reason that the complete absence of dens in this cover type during the 38-year period, despite covering 16.2% of the study area, justified eliminating alpine areas from the available habitat in the den site habitat models. Notably, the two most common den types in Scandinavia, i.e. anthill dens and open nest dens [9], both require forest habitat. In Alaska however, brown bears often hibernate in alpine areas [7, 17]. As opposed to the Alaskan bears, the Scandinavian brown bear population has a long history of sharing the landscape with a low-density but widely distributed human population, and was decimated from several thousands to only 130 individuals due to intensive harvest, including culling at the den [13, 14, 37, 59]. Compared to forests, alpine landscapes provide little cover for denning bears (H5), and the intense historical harvest may have exerted a substantial selection pressure to avoid denning in alpine habitat and other open areas. Presently, recreational cabins near the tree line are a source of high human activity in alpine areas, possibly contributing to the preference of Scandinavian bears to den in the forest. In fact, in Alaska, increased human access over the past decades has led to concerns about recreational human activity affecting high-quality brown bear denning habitat [23].

The brown bear males seemed to avoid denning in close proximity to main roads, but the selected model included no effect of forest roads, houses or cabins, i.e. only partly supporting our hypothesis of avoidance of human structures (H6). Any avoidance of inhabited houses may have been masked by the preference for high elevation, as most of the houses are concentrated along the valley bottoms. Even though cabins are concentrated in the tree line areas, they are used mostly for recreational activities in the alpine areas, which are not used as denning habitat. This may explain why cabin density did not seem to affect brown bear den site selection in Hedmark during our study period. However, the number of recreational cabins in Hedmark has increased by 31% since 2001. Of the dens in the current study, 80% were within five municipalities in which recreational cabins, mostly near alpine areas, already during our study period outnumbered the inhabited houses. Further cabin development in such areas could potentially interfere with prime brown bear denning habitat. The effect of distance to main road was quadratic, but there was little available forest habitat and very few dens more than 10 km from main roads (Fig 3F). In the population core, bears have been found to avoid denning near intermediate-sized roads with relatively high traffic intensity and easy access, but not the largest road types, possibly because intermediate-sized roads transport humans into the bear habitat, whereas larger highways provide transportation through the habitat [47]. In the current study, “main roads” include national highways, which transport people largely through the study area, but also county roads that connect human settlements within the study area. Our data did not allow us to evaluate whether bears responded differently to these two road types. Forest roads provide access to more remote areas than main roads, but in the population core, the smallest road types were argued to be largely irrelevant to denning bears as they were unplowed in winter [47]. In our study area, some forest roads are plowed in winter whereas others are not, and which ones are plowed can vary from winter to winter. Elfström and Swenson [48] found that abandoned dens in Sweden were located closer to plowed roads than dens used throughout the denning period, suggesting that such roads are indeed a source of disturbance. However, even though lack of plowing would restrict human access during much of the denning period, the den site selection happens in fall, generally before roads become inaccessible. Indeed, the period of den entry overlaps with the human moose harvest, which represents a period of higher levels of human disturbance and use of forest roads. In such a variable environment, the disturbance potential of a forest road would be hard to predict for bears selecting a den site. Den site selection based on future rather than present potential for human disturbance would require a capacity for prospective thinking that has only been studied in a small number of non-human animal taxa [69, 70].

The odds for finding a bear den decreased with increasing distance to the population core. This is consistent with the hypothesized preference for males to stay close to areas where females can be found (H7), but it could also be explained by the fact that probably all the denning bears had dispersed from this core area. However, mean dispersal distance reported for Scandinavian brown bear males is 120 km [60] and the mean distance to the edge of the population core from the random positions used in our habitat models was 130 km. Furthermore, low-density natal areas, such as the edge of the southern core area adjacent to our study area, are associated with longer dispersal distances [60]. Hence, all of Hedmark should be available for males dispersing west from the southern core area. In Slovenia as well as in our study area, researchers have observed directed movement of male brown bears from the male-biased population periphery toward the core areas during the mating season, allowing males at the periphery to take part in reproduction [36, 42]. This is consistent with the roam-to-mate hypothesis [42, 61], and the energy costs associated with this movement may be compensated by a combination of reduced resource competition and access to sheep in the Norwegian periphery. Even in Sweden without access to sheep, young brown bear males in low-density peripheral areas were found to be heavier than those inside the population core where competition would have been higher, [51]. Moreover, summer diets that include meat have been associated with higher body weights in male grizzlies [71]. In fact, males in our study area have been found to spend a disproportionate amount of time in Sweden during the mating season (May-June) and in Norway during the rest of the summer (July-August) [42]. Hence, males dispersing west from the population core may prefer to settle in the periphery where competition is low and sheep are available, but relatively close to the core areas where they can get access to females during the mating season (H7). This could potentially be limiting the pace of the westward expansion of the male segment of the Scandinavian brown bear population.

Our non-invasive method for locating den sites may give a detection bias towards areas of easy human access, near roads or settlements. However, the locations of first detection were farther from forest roads and in areas with lower densities of houses and cabins than expected from the available habitat, suggesting that the habitat characteristics of the locations of first detection reflected the habitat choice of the bears rather than the detection methods.

Like all habitat selection studies, our findings are constrained by the extent of the study area and the resolution of the data [72], as well as the set of predictors that we chose to examine. All inference of selection from our models is based on the assumption that the above-mentioned constraints allow a realistic representation of the bears’ actual den site preferences. Furthermore, there may be selection happening at different scales, influenced by factors not considered in the current study and constrained by access to hibernacula, e.g. anthills and skirt spruces which are not found in alpine areas. The insulating properties of the den and the potential for human disturbance may both be influenced by microhabitat characteristics such as horizontal and vertical cover; potentially affecting concealment and snow cover [73]. Investigating the effect of such microhabitat characteristics would require field data collected at den and control sites to supplement the currently available land cover data. Concealment cover on the microhabitat scale has been found to influence selection of day beds in Scandinavian brown bears [74], and the same may be the case for the selection of winter dens. Hence, combining landscape data with microhabitat den site characteristics would be a natural continuation from this study.

The brown bear has been functionally extinct in Norway and is currently listed as endangered [37, 75]. At the current bear densities, access to suitable denning habitat is unlikely to be a limiting factor as generally similar habitat is supporting much higher bear densities in Sweden. While avoiding certain habitat characteristics, bears in the low-density periphery should be able to find suitable denning habitat elsewhere. However, we argue that further development of recreational cabins in high-elevation forest areas may potentially interfere with prime brown bear denning habitat. Our study highlights the dynamics in which brown bear males denning at the western expansion front of the Scandinavian brown bear population move between these low-density peripheral areas and the higher-density core areas, probably trading off between low resource competition and access to sheep in the west and mating opportunities in the east.

## Supporting information

S1 Fig Number of dens per year. (TIF)

S1 Table Model selection process for den site habitat models. (DOCX)

S2 Table Model selection process for location of first detection. (DOCX)

S3 Table Summary of selected model for location of first detection. (DOCX)
