# Supplementary figures and images for: Den site selection by male brown bears at the population’s expansion front
Source: PLoS One. 2018 Aug 30;13(8):e0202653. doi: 10.1371/journal.pone.0202653 (PMC6116945; doi:10.1371/journal.pone.0202653)

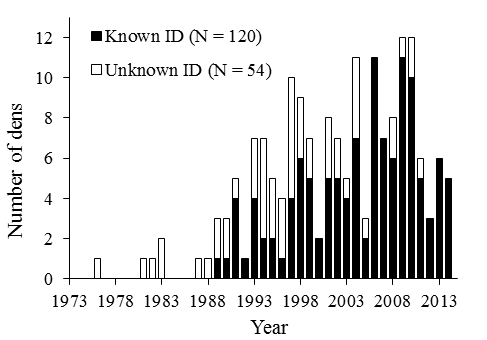

Supplement: S1 Fig — (TIF) [file pone.0202653.s001.tif]
