# Supplementary material for: Den site selection by male brown bears at the population’s expansion front
Source: PLoS One. 2018 Aug 30;13(8):e0202653. doi: 10.1371/journal.pone.0202653 (PMC6116945; doi:10.1371/journal.pone.0202653)
Supplement: S1 Table — (DOCX) [file pone.0202653.s002.docx]

**S1 Table. Model selection process for den site habitat models.** Logistic GLMMs comparing 120 den locations from 62 identified male bears with 1000 random points in Hedmark County. We used a drop 1 stepwise model selection procedure based on the Akaike Information Criterion (*AIC*). Among the best fit models (*ΔAIC* < 2), we selected the one with the smallest number of covariates. Grey fields indicate covariates that were included in the respective model, and the selected model is shown with bold letters. Covariates are RE, residual elevation; Sl, slope; A, aspect; Ru, ruggedness; SR, maximum potential solar radiation; MRd distance to main road; FRd, distance to forest road; HD, house density; CD, cabin density; PC, distance to population core areas. Sq indicates quadratic terms. Bear ID was included as a random predictor in all the models. Asterisks indicate estimates for which the 95% CI did not encompass zero. Further information about the covariates is given in Table 1, and model summary for the selected model (M9) is given in Table 2.

|  | Covariates | | | | | | | | | | | | | | | |  |  |
| --- | --- | --- | --- | --- | --- | --- | --- | --- | --- | --- | --- | --- | --- | --- | --- | --- | --- | --- |
| Mod | RE | SqRE | Sl | SqSl | A | Ru | SqRu | SR | SqSR | MRd | SqMRd | FRd | SqFRd | HD | CD | PC | *AIC* | *ΔAIC* |
| M0 |  |  |  |  |  |  |  |  |  |  |  |  |  |  |  |  | 765.9 | 154.8 |
| M1 | * | * |  |  |  |  |  |  |  | * | * |  |  |  |  | * | 619.6 | 8.5 |
| M2 | * | * |  |  |  |  |  |  |  | * | * |  |  |  |  | * | 616.9 | 5.8 |
| M3 | * | * |  |  |  |  |  |  |  | * | * |  |  |  |  | * | 614.9 | 3.8 |
| M4 | * | * |  |  |  |  |  |  |  | * | * |  |  |  |  | * | 613.3 | 2.2 |
| M5 | * | * |  |  |  |  |  |  |  | * | * |  |  |  |  | * | 612.9 | 1.8 |
| M6 | * | * |  |  |  |  |  |  |  | * | * |  |  |  |  | * | 611.1 | 0.0 |
| M7 | * | * |  |  |  |  |  |  |  | * | * |  |  |  |  | * | 611.1 | 0.0 |
| M8 | * | * | * |  |  |  |  |  |  | * | * |  |  |  |  | * | 611.3 | 0.2 |
| **M9** | ***** | ***** | ***** |  |  |  |  |  |  | ***** | ***** |  |  |  |  | ***** | **612.7** | **1.6** |
