# Supplementary material for: Den site selection by male brown bears at the population’s expansion front
Source: PLoS One. 2018 Aug 30;13(8):e0202653. doi: 10.1371/journal.pone.0202653 (PMC6116945; doi:10.1371/journal.pone.0202653)
Supplement: S2 Table — (DOCX) [file pone.0202653.s003.docx]

S2 Table. Model selection process for location of first detection. Logistic GLMs comparing locations of first detection of 165 brown bear dens with 1000 randomly generated points within Hedmark County. We used a drop 1 stepwise model selection procedure based on the Akaike Information Criterion (AIC). Among the best fit models (ΔAIC < 2), we selected the one with the smallest number of covariates. Grey fields indicate covariates that were included in the respective model, and the selected model is showed with bold letters. Covariates are RE, residual elevation; Sl, slope; Ru, ruggedness; MRd, distance to main road; FRd, distance to forest road; HD, house density; CD, cabin density. Sq indicates quadratic terms. Asterisks indicate estimates for which the 95% CI did not encompass zero. Further information about the covariates is given in Table 1, and model summary for the selected model (M.det3) is given in S3 Table.

|  | Covariates | | | | | | | |  |  |
| --- | --- | --- | --- | --- | --- | --- | --- | --- | --- | --- |
| Mod | RE | SqRE | Sl | Ru | MRd | FRd | HD | CD | *AIC* | *ΔAIC* |
| M.det0 |  |  |  |  |  |  |  |  | 952.44 | 77.30 |
| M.det1 |  | ***** |  |  |  | ***** | ***** | ***** | 878.11 | 2.97 |
| M.det2 |  | ***** |  |  |  | ***** | ***** | ***** | 876.15 | 1.01 |
| **M.det3** |  | ***** |  |  |  | ***** | ***** | ***** | **875.14** | **0.00** |
