# Supplementary material for: Den site selection by male brown bears at the population’s expansion front
Source: PLoS One. 2018 Aug 30;13(8):e0202653. doi: 10.1371/journal.pone.0202653 (PMC6116945; doi:10.1371/journal.pone.0202653)
Supplement: S3 Table — (DOCX) [file pone.0202653.s004.docx]

**S3 Table. Summary of selected model for location of first detection.** Logistic GLM (M.det3, S2 Table) comparing locations of first detection of 165 male brown bear dens with 1000 randomly generated points within Hedmark County. Further information about the covariates is given in Table 1, and the model selection process is shown in S2 Table.

| Covariates | Estimate | *SE* | *Z* | *P* |
| --- | --- | --- | --- | --- |
| Residual elevation | 0.21 | 0.74 | 0.28 | 0.781 |
| Residual elevation^2 | 7.38 | 3.04 | 2.43 | 0.015 |
| Slope (log(x+1)) | 0.34 | 0.12 | 2.86 | 0.004 |
| Dist. to forest road (log(x+1)) | -1.32 | 0.28 | -4.65 | <0.0001 |
| House density (log(x+1)) | -1.09 | 0.21 | -5.11 | <0.0001 |
| Cabin density (log(x+1)) | -0.62 | 0.21 | -2.92 | 0.004 |
